# Differential expression of CD8 defines phenotypically distinct cytotoxic T cells in cancer and multiple sclerosis

**DOI:** 10.1002/ctm2.1068

**Published:** 2022-12-11

**Authors:** Tobias Burkard, Martina Herrero San Juan, Caroline Dreis, Anastasiia Kiprina, Dmitry Namgaladze, Kai Siebenbrodt, Sebastian Luger, Christian Foerch, Josef M. Pfeilschifter, Andreas Weigert, Heinfried H. Radeke

**Affiliations:** ^1^ Pharmazentrum Frankfurt/ZAFES Institute of Pharmacology and Toxicology Hospital of the Goethe University Frankfurt am Main Germany; ^2^ Faculty of Medicine Institute of Biochemistry I Goethe‐University Frankfurt/Main Frankfurt am Main Germany; ^3^ Department of Neurology Goethe University Hospital Frankfurt Frankfurt am Main Germany; ^4^ Frankfurt Cancer Institute Goethe‐University Frankfurt Frankfurt Germany; ^5^ Cardio‐Pulmonary Institute (CPI) Frankfurt Germany; ^6^ Epilepsy Center Frankfurt Rhine‐Main Department of Neurology University Hospital Frankfurt, Frankfurt, Germany

**Keywords:** CD8^+^ T cells, mTOR, multiple sclerosis, multispectral imaging, tumour immunity

## Abstract

**Background:**

Cytotoxic T lymphocytes take on a leading role in many immune‐related diseases. They function as key effector immune cells fighting cancer cells, but they are also considerably involved in autoimmune diseases. Common to both situations, CD8^+^ T cells need to adapt their metabolism and effector function to the harsh and nutrient‐deprived conditions of the disease‐associated microenvironment.

**Methods:**

We used an in vitro starvation as well as rapamycin treatment protocol mimicking nutrient deprivation to generate CD8^Low^ versus CD8^High^ T cells and performed FACS‐Sorting followed by transcriptomic profiling of the cytotoxic T cell subsets. Prominent markers identified in the CD8^Low^ versus the CD8^High^ T cells were then used to investigate the presence of these cell subsets in immune‐related human diseases. Employing cancer tissue microarrays and PhenOptics multispectral imaging as well as flow cytometry, we studied these CD8^+^ T cell subsets in cancer and relapsing‐remitting multiple sclerosis patients.

**Results:**

Starvation induced a decreased expression of CD8, yielding a CD8^Low^ T cell subpopulation with an altered transcriptomic signature and reduced effector function. CD8^Low^ T cell showed enhanced ST2L and IL6ST (CD130) expression compared to CD8^High^ T cells which expressed elevated KLRD1 (CD94) and granzyme B levels within the tumour microenvironment (TME). Spatial analysis revealed the presence of CD8^High^ T cells in close proximity to tumour cells, while the CD8^Low^ T cells resided at the tumour boundaries. Importantly, the number of tumour‐infiltrating CD8^Low^ T lymphocytes correlated with a poor prognosis as well as with enhanced cancer progression in human mammary carcinoma. We also found a reduced frequency of CD8^Low^ T lymphocytes in a cohort of relapse (disease active) multiple sclerosis patients compared to healthy subjects during immune cell starvation in vitro.

**Conclusions:**

In summary, our data show that functionally distinct cytotoxic T lymphocytes can be identified based on their expression of CD8. Indicating a more general role in CD8 T cell immunity, these cells may play opposing roles in the TME, and also in the pathophysiology of autoimmune diseases such as multiple sclerosis.

## Graphical abstract

Transcriptome analysis and functional characterisation reveals distinct phenotypes of human CD8 ^Low^‐ and high‐expressing cytotoxic T cells.

CD8^Low^ T cells versus CD8^High^ T cells show divergent localisation patterns in tumours and are associated with patient prognosis.

Reduction in CD8^Low^ compared to CD8^High^ T cells in RRMS patients experiencing relapse.

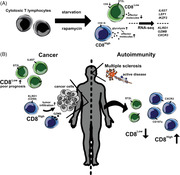

## 1 BACKGROUND

The response of cytotoxic CD8^+^ T lymphocytes is found to be considerably dysregulated in various pathological conditions including cancer and autoimmune diseases. Within the nutrient‐deprived TME, lymphocytes undergo metabolic stress, which can eventually result in impaired effector function.^1^ The relevance of such an impaired effector program is exemplified by the success of immune checkpoint blockade that can reactivate the immune effector response for fighting cancer progression.^2^ However, the understanding of the phenotypic changes due to metabolic adaptation of T lymphocytes is still limited and needs to be improved in order to increase the therapeutic benefit of current immunotherapy in cancer patients.

The mechanistic target of rapamycin (mTOR) signalling is an essential metabolic pathway that shapes T cell differentiation and cell survival and which also functions as a molecular sensor for nutrients.^3^ Under conditions of metabolic stress; for example, during cell starvation, mTORC1 activity is decreased, which eventually induces autophagy.^4^ Rapamycin as an allosteric mTORC1 inhibitor and immunosuppressive drug has been described to drive a regulatory phenotype, notably FOXP3 expression in T cells,^5^, ^6^, ^7^ but it has also been shown to favour the differentiation of memory T cells.^8^ While the mTORC1 activity is tightly linked to a glycolytic effector T cell response in CD8^+^ T cells,^9^ mTORC1 inhibition as a result of an nutrient‐deprived TME can eventually impede the anabolic metabolism of lymphocytes that is required to exert potent effector function.^1^, ^10^ Of note, the targeting of the mTOR pathway by rapamycin has already been discussed as a therapeutic approach in relapsing‐remitting multiple sclerosis (RRMS), which is the most common form of this demyelinating autoimmune disease and which aims modulate T cell immune responses in order to prevent relapses.^11^, ^12^

The CD8 molecule expressed by cytotoxic T lymphocytes functions as an important co‐receptor of the T cell receptor (TCR) for the recognition of MHC class I molecules, which is closely related to cell activity.^13^ We previously observed CD8 downregulation on cytotoxic T lymphocytes under in vitro starvation conditions, which was correlated with the capacity of CD8^Low^ to suppress the proliferation of responder T cells.^14^, ^15^ In the current study, we performed a deep characterisation of the CD8^Low^ versus the CD8^High^ T cells to generate new insight into potential divergent phenotypic and functional properties, and to evaluate their presence in relevant human disease settings, i.e. in solid tumours and RRMS.

## 2 MATERIAL AND METHODS

### 2.1 Buffy coats

PBMC and CD8^+^ T lymphocytes were purified for both the in vitro experiments and the transcriptome analysis of CD8^Low^ and CD8^High^ subsets from buffy coats drawn from commercially‐available anonymous healthy donors of the blood donation centre DRK‐Blutspendedienst Baden‐Württemberg‐Hessen, Institut für Transfusionsmedizin und Immunhämatologie Frankfurt am Main, Frankfurt, Germany.

### 2.2 PBMC isolation and serum starvation protocol

Human peripheral blood mononuclear cells (PBMC) were enriched from EDTA blood by density gradient centrifugation using a Ficoll‐Histopaque 1.077 g/ml density (Sigma‐Aldrich, Steinheim, Germany). PBMC were seeded at a density of 1 × 10^6^ cells/ml into 6‐well plates (Greiner bio‐one, Frickenhausen, Germany) and were cultivated for 40 h of serum starvation in RPMI 1640 + Glutamax supplemented with 50 mM β‐mercaptoethanol, 1 mM sodium pyruvate, 100 μg/ml streptomycin and 100 IU/ml penicillin (all from Thermo Fisher Scientific, Waltham, MA) and 2 nM Hepes (Sigma‐Aldrich, Steinheim, Germany). For all FACS sorting experiments, the CD8^+^ T lymphocyte were pre‐enriched from PBMC by negative immunomagnetic selection using the EasySep™ Human CD8^+^ T cell Isolation Kit (Stemcell Technologies, Vancouver, Canada) according to the manufacturer's instructions (Supplementary Figure S1) and cultured at a density of 5 × 10^5^ cells and as described above. For mTORC1 inhibition, the CD8^+^ T lymphocytes were treated with a rapamycin concentration range starting from 100 ng/ml [dissolved in dimethylsulphoxide (DMSO), LC Laboratories, Woburn, MA] in cell culture medium supplemented with 10% autologous serum.

### 2.3 Cell sorting for the CD8^+^ T cell subset characterisations

CD8^+^ T lymphocytes were purified from human buffy coats as described in Sections 2.1 and 2.2 and the cells were sorted according to their CD8 expression (anti‐CD8‐V450, clone: RPA‐T8, BD Biosciences, Heidelberg, Germany, RRID: AB_1645581) into CD8^Low^ and CD8^High^ T cell subsets with the FACSymphony S6 Cell sorter (BD Biosciences, San Diego, CA). A general gating strategy for the CD8^+^ T lymphocytes (Supplementary Figure S2A) is provided in the supporting information. As described elsewhere^14^, ^15^ the sorting samples were compared to a donor‐matching 10 % autologous serum control (Supplementary Figure S2B and C).

### 2.4 CD8 expression stability on T cell subsets after cell sorting

To test the stability of the CD8 expression on the T cell subsets, 1 × 10^5^ CD8^Low^ or CD8^High^ sorted T lymphocytes were cultured for 24 and 48 h together with anti‐CD3/28/2 antibody complexes (Stemcell Technologies, Vancouver Canada) and subsequently analysed for CD8 expression (anti‐CD8‐V450, clone: RPA‐T8, BD Biosciences, Heidelberg, Germany, RRID: AB_1645581). The samples were measured with a FACS Canto II flow cytometer (BD Biosciences, San Diego, CA). The data were analysed using FlowJo software V10.7.1 (BD Life Sciences).

### 2.5 Metabolic measurement of CD8^+^ T cell subsets

The sorted CD8^+^ T lymphocyte subsets were seeded into 8‐well Seahorse XF HS Miniplates (Agilent, Santa Clara, CA) and incubated with 25 μl/ml anti‐CD3/28/2 T cell activation antibody cocktail (Stemcell Technologies, Vancouver, Canada). To improve the adherence of the T cells, plate wells were coated with Cell‐Tak Cell Tissue Adhesive (Corning, Corning, New York, NY) at a concentration of 22.4 μg/ml according to manufacturer's instructions. After 24 h, the medium was replaced with the assay medium XF Base media (Agilent, Santa Clara, CA) with glucose (10 mM) and L‐glutamine (2 mM), pH 7.4 at 37 °C (all supplements by Thermo Fisher Scientific, Waltham, MA). Subsequent to equilibration, as a measure of glycolysis, ECAR was measured during a Seahorse Mito Stress assay (Agilent, Santa Clara, CA) with a first addition of oligomycin (2.5 μM), followed by carbonyl cyanide 4‐(trifluoromethoxy) phenylhydrazone (FCCP; 1 μM), rotenone (0.25 μM) and antimycin A (10 μg/ml). All values were normalised to protein concentration using the DC Protein Assay Kit (Bio‐Rad Laboratories, Hercules, CA) according to the manufacturer's instructions.

### 2.6 Intracellular cytokine staining (ICCS) of CD8^+^ T cell subsets

CD8^+^ T cells were sorted and cultured as described in Sections 2.2 and 2.3. For the intracellular measurement of interferon‐γ (IFN‐γ), granzyme B (GZMB) and perforin‐1 (PRF‐1), the CD8^+^ T cell subsets were incubated with 3 μg/ml brefeldin A (Thermo Fisher Scientific, Waltham, MA) for 6 h. The harvested cells were then stained with anti‐CD8‐V450 (for details see above), anti‐IFN‐γ‐Alexa Fluor 488 (clone: B27, RRID:AB_396827), anti‐granzyme B‐Alexa Fluor 647 (clone: GB11, RRID:AB_10897997) and anti‐perforin‐1‐PE‐CF594 (clone: δG9 RRID:AB_2738410), all obtained from BD Biosciences (San Diego, CA) using the BD Cytofix/Cytoperm™ Fixation/Permeabilization Kit (BD Biosciences San Diego, CA) according to the manufacturer's instructions. All antibodies were titrated to determine the optimal concentration for use. The stained samples were acquired with a FACSymphony™ A5 Cell Analyzer (BD Biosciences, Heidelberg, Germany). The FMO stainings were used for gating. The data were analysed using FlowJo software V10.7.1 (BD Life Sciences).

### 2.7 RNA sequencing (RNA‐seq) of CD8^Low^ and CD8^High^ T lymphocytes

mRNA‐isolation from a minimum of 1 × 10^5^ cells of the CD8^+^ T cell subsets was performed immediately after cell sorting using the RNeasy Micro Kit (Qiagen, Hilden, Germany) according to the manufacturer's instructions. RNA concentrations and the RNA integrity number (RIN) were determined with the Agilent 4150 Tape Station instrument using the Agilent High‐Sensitivity RNA Screen Tape (Agilent, Santa Clara, CA). All samples that were used for sequencing had RINe values greater than or equal to 8.8. Libraries were prepared with 14 ng RNA input per sample using the QuantSeq 3′mRNA‐Seq Library Prep Kit‐FWD and the UDI 12 nt Unique Dual Indexing Add‐on kit (both Lexogen, Vienna, Austria) according to the manufacturer's instructions. For library amplification the optimal number of cycles was assessed for each sample. The quality and concentration of the libraries was determined on an Agilent 4150 Tapestation instrument using the Agilent High Sensitivity D1000 ScreenTape (Agilent, Santa Clara, CA). The pooled cDNA libraries (libraries were diluted to 2 nM concentration) were sequenced in an Illumina NextSeq 2000 instrument (Illumina, San Diego, CA). These sequence data have been submitted to the GEO database under accession number GSE207621. The QuantSeq data analysis pipeline (Lexogen, Vienna, Austria) was used for data processing and the calculation of normalised counts.

### 2.8 Gene set enrichment analysis (GSEA)

Gene set enrichment analysis (GSEA) was performed on the normalised expression data of the CD8^Low^ and CD8^High^ T cell subsets as described.^16^ The analysis was performed on 1 September 2021 using GSEA v4.1.0 (US San Diego and Broad Institute) and Human ENSEMBL_Gene_MSigDBv7.1 platform. The enrichment was considered significant if the FDR q‐value was less than 0.25.

### 2.9 Quantitative PCR

In order to validate the differential gene expression by the CD8^Low^ and CD8^High^ T cell subsets, the mRNA that was isolated as described in Section 2.7 and was transcribed into cDNA using SuperScript™ VILO™ Master Mix (Thermo Fisher Scientific, Waltham, MA) according to the manufacturer's instructions. To quantify the *CD8A* (Hs00233520_m1), *IKZF2* (Hs00212361_m1), *IL6ST* (Hs00174360_m1), *LEF1* (Hs01547250_m1), *SATB1* (Hs00962580_m1), *XBP1* (Hs00231936_m1), *PRF1* (Hs00169473_m1), *CXCR3* (Hs00171041_m1), *KLRD1* (Hs00233844_m1) and *GZMB* (HS00188051_m1) mRNA levels in the CD8^+^ T cells, their relative gene expression was calculated by normalisation to the housekeeping genes *GAPDH* (Hs02758991_g1) and *ACTB* (Primer Design, Southampton, UK) by using the 2^−ΔCt^ method (all probes were obtained from Applied Biosystems, Waltham, MA).

### 2.10 Multiplex immunohistochemistry and immunofluorescence analysis

For the investigation of CD8^+^ T lymphocytes in different tumour entities, the following human Tissue Microarrays (TMAs) were purchased from Biomax, USA: one breast cancer with cancer adjacent breast tissue array (catalogue: BC081116e), one endometrium cancer with endometrium tissue array (catalogue: EM1021a) and one colon cancer tissue array (catalogue: BC05012a). The TMA slides were stained with Opal 7‐Color Automation IHC Kits (Akoya Biosciences, Menlo Park, CA) using the BOND‐RX Multiplex IHC Stainer (Leica, Wetzler, Germany) and the BOND Epitope Retrieval Solutions ER1 or ER2 (Leica, Wetzler, Germany). The following antibodies were used: anti‐CD3 (Ventana Medical Systems, Tucson, AZ, 2GV6, RRID:AB_2335978), anti‐CD94 (Abcam, Cambridge, UK, EPR21003, RRID:AB_2920906), anti‐ST2L (Proteintech, Chicago, IL, 11920‐1‐AP, RRID:AB_906359), anti‐IL6ST (Sigma‐Aldrich, Steinheim, Germany, HPA010558, RRID:AB_1078439), anti‐CD8 (Agilent, Santa Clara, CA, C8/144B RRID: AB_2075537) and anti‐granzyme B (Abcam, Cambridge, UK, EPR8260, RRID:AB_2889221). In order to test the stability of the targeted antigen epitopes, FFPE tissue sections were repeatedly subjected to heat‐induced antigen retrieval, and changes in the staining signals were assessed.^17^ The staining order of antigens was selected based on this validation process. For multispectral imaging the Vectra Polaris system was used (Akoya Biosciences, Menlo Park, CA). Fluorescent scans were analysed with the Halo software (Indica Labs, Corrales, NM) using the Highplex FL, TMA, Classifier and Spatial Analysis modules to detect cytotoxic (CD3^+^ CD8^+^) T cells in the different cores. The object data for each individual core were exported and the CD8 cell intensity analysis of cytotoxic T cells was performed using FlowJo software V10.7.1 (BD Life Sciences) (Supplementary Figure S3). A summary of analysed TMA patient cores is provided in the supplementary information (Supplementary Table S1).

### 2.11 Breast cancer patients

For the FACS analysis of the CD8 subsets, human breast cancer patients (*n* = 14) from the University Cancer Center (UCT) Frankfurt study (ICD10‐Code: C50.9) were analysed and as described elsewhere.^17^ The following FACS antibodies were used for CD8 gating: anti‐CD4‐PE‐CF594 (clone: RPA‐T4, RRID: AB_11154394), CD8‐BV786 (clone: RPA‐T8, RRID: AB_2687487), CD33‐BV510 (clone: WM53, RRID: AB_2738102), CD19‐APC‐H7 (clone: SJ25C1, AB_1645468) and TCRαβ‐FITC (clone: T10B9.1A‐31, RRID: AB_10892811) all from BD Biosciences (San Diego, CA) and anti‐CD45‐AF700 (clone: 2D1, RRID:AB_2566373), CD56‐PerCP‐Cy5.5 (clone: 5.1H11, RRID: AB_2563914) and anti‐CD279‐APC (PD‐1) (clone: EH12.2H7, RRID: AB_940473), all from BioLegend, San Diego, CA. The study was approved by the Institutional Review Boards of the UCT Frankfurt and the Ethics Committee at University Hospital Frankfurt (approval numbers: SGO‐01‐2014 and SGO‐01‐2016).

### 2.12 RRMS patient samples

Peripheral venous EDTA blood was obtained from *n* = 25 patients with RRMS (*n* = 13 in relapse, *n* = 12 in remission). Patients with a relapse within 30 days before blood withdrawal were allocated to the relapse patient group, whereas all other patients were included in the analyses as being on remission. All patients met the following inclusion criteria: (i) had a diagnosis of MS with the relapsing‐remitting form of the disease, (ii) were aged over 18 years, (iii) were undergoing disease‐modifying therapy (DMT) with natalizumab, glatiramer acetate or fingolimod, (iv) were not undergoing DMT but were in relapse and (v) had no previous study participation. In addition, age and sex‐matching healthy participants served as the control group. The demographic and clinical characteristics as well as the DMTs of all the patients and participants are summarised in the Supplementary Table S2 within the supporting information. All samples were collected between October 2020 and July 2021. This research was approved by the local ethics committee of the Faculty of Medicine, Goethe‐University Frankfurt and was conducted in accordance to the declaration of Helsinki. All patients and participants gave their written informed consent.

### 2.13 Flow cytometry for RRMS study samples

For the characterisation of the surface molecules on the lymphocytes of RRMS patients or the healthy controls, a maximum of 1 × 10^6^ PBMC were stained as a single cell suspension with the following antibodies purchased from BD Biosciences (San Diego, CA): anti‐CD4‐BB630 (clone: SK3), CD3‐BUV805 (clone: SK7, RRID: AB_2870181), CD33‐BV510 (clone: WM53, RRID: AB_2738102), CD8‐BV650 (clone: RPA‐T8, RRID: AB_2744462), CD107a‐PE‐Cy5 (clone: H4A3, RRID: AB_396136) and anti‐CD56‐BV605 (clone: HCD56, RRID: AB_2561912), anti‐CD183‐APC (CXCR3) (clone: G025H7, RRID: AB_10983064), both from BioLegend, San Diego, CA and anti‐ST2L‐FITC (MD Biosciences, Zürich, Switzerland, clone: B4E6, RRID: AB_947548). The antibodies were titrated for optimal FACS staining. To avoid non‐specific antibody binding to the F_c_ receptors, the cells were incubated for 10 min at 4°C with 0.1% PBS/FCS containing human F_c_ block (BD Pharmingen, San Diego, CA). In order to exclude non‐viable cells from the FACS analysis, cells were stained using the Zombie UV™ Fixable Viability Kit (BioLegend, San Diego, CA). The samples were acquired with a FACSymphony S6 Cell sorter (BD Biosciences, San Diego, CA). Compensation beads (BD Compbeads, San Diego, CA) as well as Fluorescence Minus One (FMO) controls were used to calculate corrections for spectral overlap and to determine the spread of fluorescence for the combination of antibodies used. The flow cytometer performance was regularly controlled using Cytometer Setup and Tracking beads (BD Biosciences, San Diego, CA). The data were analysed using FlowJo software V10.7.1 (BD Life Sciences).

### 2.14 Statistical analysis

Statistical analysis was performed using GraphPad Prism 8 software (GraphPad Software Inc., San Diego, CA). For the statistical comparisons between the RRMS patients (relapse or remission group) and the healthy controls, Mann–Whitney *U* tests were applied. For paired statistical analysis of the data, the differences between groups were calculated with the Wilcoxon matched‐pairs signed‐rank test or paired *t*‐test were used after testing for normal distribution using the Shapiro–Wilk test (sample sizes below 30) or the Kolmogorov–Smirnov test (sample sizes above 30). For multiple comparisons, the Friedman test was used. *p* Values below .05 denoted statistical significance (n.s. for *p* > .05, * for *p* ≤ .05, ** for *p* < .01, *** for *p* < .001 and **** for *p* < .0001).

## 3 RESULTS

### 3.1 Starvation induces stable CD8 downregulation on cytotoxic T cells

**FIGURE 1:**
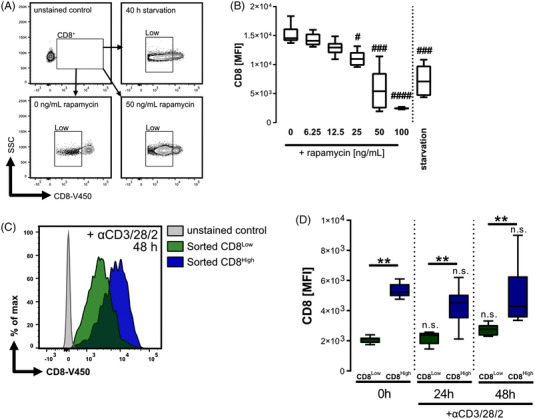
mTOR‐dependent starvation induces CD8^Low^ T cells. (A, B) Purified human cytotoxic T lymphocytes were cultured either under serum withdrawal or were treated with different rapamycin concentrations in the presence of 10 % autologous serum for 40 h. CD8α surface expression (V450), mean fluorescence intensity (MFI) was analysed by flow cytometry. (A) Representative FACS plots for the gating of low‐CD8 expressing T cells after starvation or rapamycin treatment (B) Data are obtained from *n* = 8 donor samples. # indicate multiple comparisons to the untreated DMSO control (0 ng/ml), using Friedman's test with Dunn's post‐test. #*p* ≤ .05, ###*p* < .001, ####*p* < .0001 (C, D) Cytotoxic T lymphocytes were sorted according to their CD8 surface expression into low‐ and high‐expressing T lymphocytes and separately cultured for 48 h. (C) Representative flow cytometry histogram showing the CD8 (V450) expression of sorted CD8^Low^ (green) and CD8^High^ (blue) T cell subpopulations after 48 h of anti‐CD3/28/2 TCR activation bead treatment. (D) Comparison of CD8 expression (MFI) for the two sorted subpopulations after 0 h (*n* = 10 donors), 24 h (*n* = 10 donors) and 48 h (*n* = 9 donors) using Wilcoxon matched‐pairs signed rank test. **p < .01. Multiple comparisons (Dunn's post‐test) of the 0 h with 24 and 48 h time points were not significant (n.s.). (B, D) The box plots mark the 5th percentile, the median and 95th percentile

The mTOR pathway centrally controls the metabolism of T cells. A number of disease‐related conditions in the TME and inflammatory environments may lead to an inhibition of mTOR in the immune cells.^10^ Therefore, we studied human cytotoxic T lymphocyte behaviour under the conditions of serum starvation or treatment with the mTORC1 inhibitor rapamycin (‘pseudo‐starvation’). Cytotoxic T lymphocytes showed decreased expression of CD8α 40 h after serum starvation as well as in a dose‐dependent manner after treatment with rapamycin (Figure 1A and B). We subsequently isolated CD8^Low^ and CD8^High^ T cells by FACS‐Sorting in order to investigate whether the differential CD8 expression was dynamic or stable. The CD8 expression on T cells is altered during T cell activation.^13^ Therefore, the cytotoxic T lymphocytes were treated for 48 h with an anti‐CD3/28/2 antibody cocktail to mimic the T cell receptor (TCR) activation. We subsequently reassessed CD8α expression in both the sorted CD8^Low^ and CD8^High^ subpopulations after 24 and 48 h and found that CD8 surface expression remained stable for 48 h even after TCR stimulation (Figure 1C and D).

### 3.2 CD8^Low^ T cells show reduced cytotoxic effector function

**FIGURE 2:**
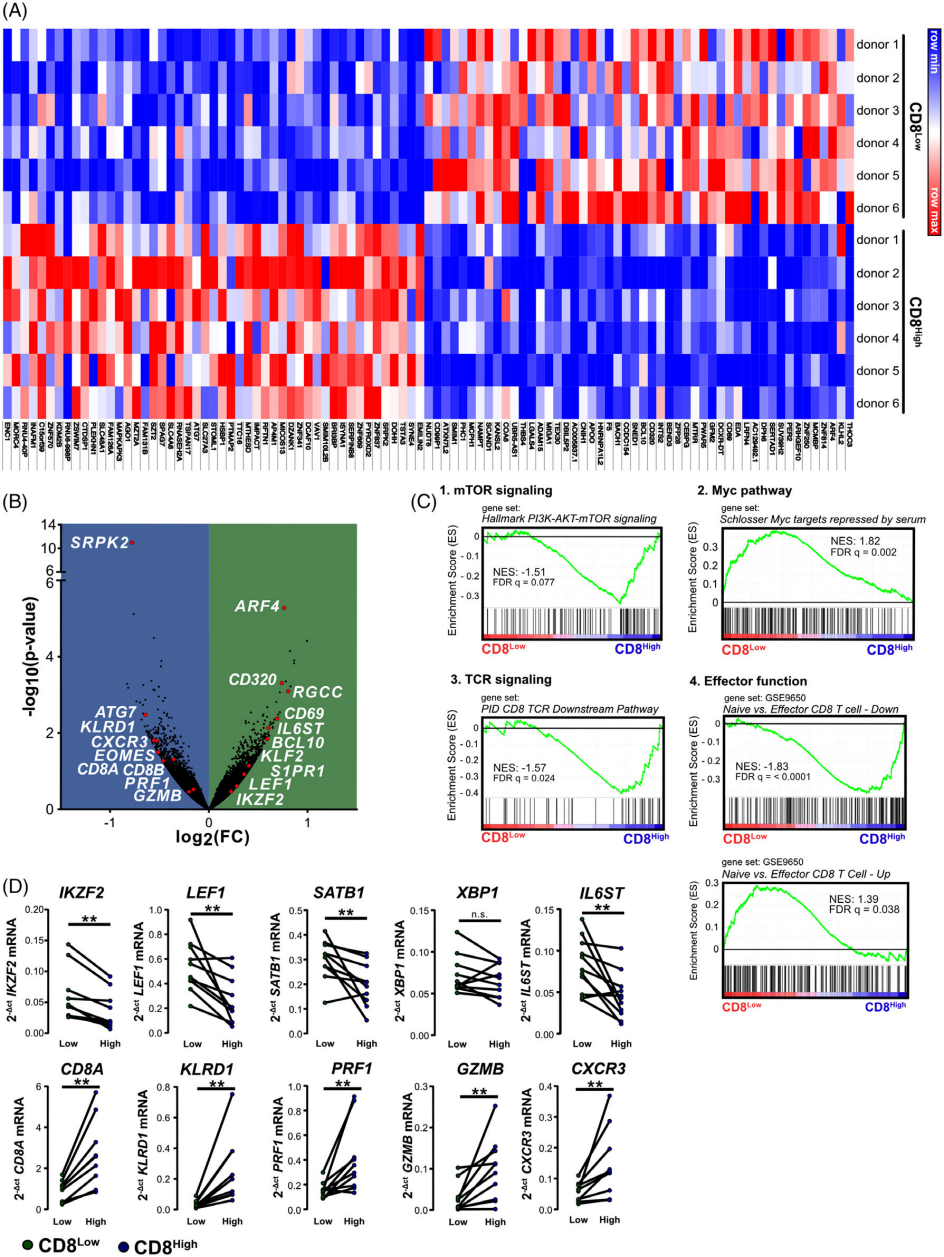
Transcriptional analysis indicates functional diversity of sorted CD8^Low^ and CD8^High^ subpopulations. Cytotoxic T cells were starved for 40 h before sorting into CD8^Low^ and CD8^High^ subpopulations and subsequent mRNA isolation for transcriptional profiling. (A) Heat‐map of the top 50 genes in both CD8 subsets. Red indicates high expression and blue indicates low expression. (B) Volcano plot of relative difference in all transcripts (*n* = 13,864) obtained from *n* = 6 donors. Some genes of interest for hypothesis generation were listed for CD8^Low^ (green background) and CD8^High^ (blue background). (C) Representative plots of the GSEA analysis for CD8^Low^ (red) and CD8^High^ (blue) T lymphocytes of *n* = 6 donors. The normalised enrichment score (NES) and false‐discovery rate (FDR) *q*‐value for the tested gene set are indicated. (D) Transcripts of interest for defining CD8^Low^ and CD8^High^ T cells were validated from normalised counts of RNA‐seq data by qPCR of sorted CD8^Low^ (green bars) and CD8^High^ (blue bars) obtained from *n* = 9–11 different donor samples. n.s. not significant, **p < .01, using Wilcoxon matched‐pairs signed rank test

To better characterise the phenotype of these stable T cell populations, we performed whole transcriptome analysis of both sorted CD8^Low^ and CD8^High^ T lymphocytes. As a result of high donor heterogeneity, we observed few significant differential expression of transcripts in both lymphocyte subsets (Figure 2A and B). However using GSEA on the resulting datasets to investigate T cell‐specific transcriptional programs in CD8^Low^ compared to CD8^High^ T cells, revealed significantly altered common CD8^+^‐related pathways and metabolically related T cell states (Figure 2C). First, we found an enrichment of transcripts that were related to an active PI3K‐AKT‐mTOR signalling pathway in the CD8^High^ T cells (Figure 2C). This strengthens the notion that the mTOR pathway functions as a metabolic sensor in cytotoxic T cells, but also shows that mTOR signalling is restrained in CD8^Low^ T cells during serum starvation. Besides mTOR, the transcription factor MYC is an additional major controller of the metabolism in T lymphocytes.^18^ We found an enrichment of MYC target genes that were repressed by serum starvation in our CD8^Low^ T lymphocytes. Importantly, transcripts that were related to TCR downstream signalling were enriched in the CD8^High^ T lymphocyte subset (Figure 2C). Accordingly, two gene sets for cytotoxic effector function indicate a higher effector function in the CD8^High^ T cell subset as well. In contrast, we were able to validate that CD8^Low^ T cells showed enhanced expression of markers associated with regulatory‐like T cell function, among them transcription factors that orchestrate the transcriptional switch towards T_reg_ cells and maintain lineage commitment.^19^ We found higher expression of the transcription factor Helios (*IKZF2*) in CD8^Low^ T cells as well as additional transcription factors such as *SATB1* and *LEF1*, thus indicating that CD8^Low^ T cells may have the potential towards T_reg_ cell differentiation (Figure 2D). Additionally, we validated differentially expressed transcripts such as *IL6ST*, which encodes for a subunit of the interleukin 6 receptor (CD130) and which was highly expressed in the CD8^Low^ T cells (Figure 2D). By contrast, *KLRD1*, encoding a killer cell lectin‐like receptor (CD94), as well as *GZMB* and *PFR1*, encoding the cytotoxic effector molecules granzyme B and perforin‐1, were increased in the CD8^High^ T lymphocytes. Altogether, these data support a pronounced cytotoxic effector function in CD8^High^ T cells. Thus, distinct CD8 expression by cytotoxic T lymphocytes appears to mark two discrete phenotypes upon starvation. Moreover, we observed that tendencies in differential expression of normalised RNA‐seq count and the subsequent qPCR validation studies did correlate. While CD8^Low^ T lymphocytes showed a regulatory‐like phenotype, the CD8^High^ appear to be the primary cytotoxic effectors. Of note, the expression of *CD8A* was reduced in CD8^Low^ compared to the CD8^High^ T lymphocyte subpopulation, indicating that the stable suppression of CD8 expression upon serum starvation occurs at the transcriptional level (Figure 2D).

### 3.3 Effector CD8^High^ T lymphocytes are characterised by an increased anabolic potential

**FIGURE 3:**
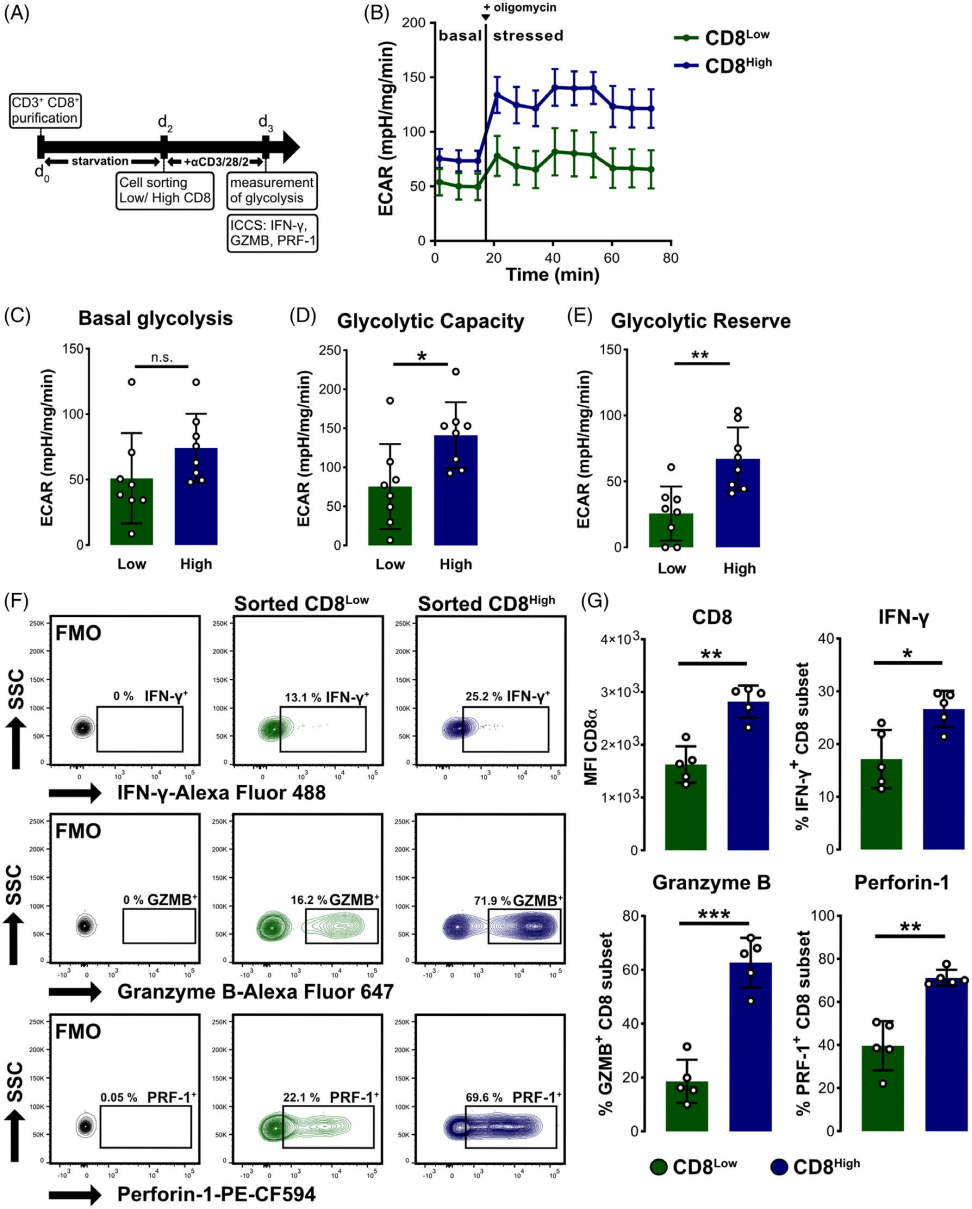
Distinct metabolic activity and glycolytic profiling of activated CD8^Low^ and CD8^High^ T cell subpopulations. (A) CD8^+^ T lymphocytes were starved as previously described before being sorted into CD8^Low^ (green) and CD8^High^ (blue) T cell subpopulations. Subsequently, the T cell subsets were stimulated for 24 h with anti‐CD3/28/2 TCR activation antibodies before measurement in the mitochondrial stress test assay or being subjected to intracellular cytokine staining (ICCS). (B–E) Extracellular acidification rate (ECAR) was normalised to protein content. Data were obtained from *n* = 8 donors. (B) Measurement of glycolysis over the time. Data are shown as mean ± SEM. (C) Basal glycolysis calculated as mean of three values before oligomycin was added. (D) Glycolytic capacity was calculated from the maximal ECAR rate reached following the addition of oligomycin (after 21 min). (E) The glycolytic reserve was calculated as difference between the glycolytic capacity and basal glycolysis. (C–E) Data expressed as mean ± SD. *p ≤ .05, **p ≤ .01, n.s., not significant, using Wilcoxon matched‐pairs signed rank test. (F) Representative contour plots for IFN‐γ (Alexa Fluor 488), Granzyme B (Alexa Fluor 647) and Perforin‐1 (PE‐CF594) expression by sorted CD8^Low^ and CD8^High^ T cell subsets. (G) Mean fluorescence intensity (MFI) of sorted CD8^Low^ and CD8^High^ as well as percentages of IFN‐γ^+^, Granzyme B^+^ and Perforin‐1^+^ cells from *n* = 5 donor samples, mean ± SD, *p ≤ .05, **p ≤ .01, ***p ≤ .001 using the paired *t*‐test.

Effector function as well as the activation of the cytotoxic CD8^+^ T cell requires a metabolic switch towards anabolic pathways.^20^ As the GSEA analysis of the CD8^Low^ and CD8^High^ T lymphocytes indicated an alteration in metabolism‐related mTOR and MYC pathways, we wondered if the metabolic potential of these two CD8^+^ T cell subpopulations differs. Thus, we sorted CD8^Low^ and CD8^High^ T lymphocytes and TCR‐activated them, followed by measurement of their glycolysis (Figure 3A). We used the maximal extracellular acidification rates (ECAR) as an indicator of glycolysis and assessed the functional capacity of both subsets (Figure 3B). While the basal glycolysis rate appeared to be only slightly different between the low and high CD8‐expressing T cell subsets (Figure 3C), we found an increased glycolytic capacity (maximal ECAR rate) in the CD8^High^ T cells (Figure 3D). Notably, the glycolytic reserve, in particular, seemed to be higher in the CD8^High^ T cells compared to the CD8^Low^ subset (Figure 3E), thus indicating an overall higher bioenergetic demand driving glycolysis, which is eventually linked to the increased effector function of the cytotoxic T lymphocytes. Importantly, in line with increased glycolytic demand in CD8^High^ T cells, these cells expressed higher levels of IFN‐γ, granzyme B (GZMB) and perforin‐1 (PRF‐1) revealed by intracellular FACS analysis (Figure 3F and G). Of note, during the in vitro co‐culture with human MCF‐7 breast cancer cells, we observed a higher allogenic T cell cytotoxicity of the CD8^High^ T cells (Supplementary Figure S5). Taken together, our data suggest that the adapted metabolism of CD8^+^ cytotoxic T lymphocytes due to metabolic stress impacts T cell differentiation and effector function.

### 3.4 IL6ST and ST2L mark CD8^Low^, while granzyme B and KLRD1 mark CD8^High^ T cells in cancer

**FIGURE 4:**
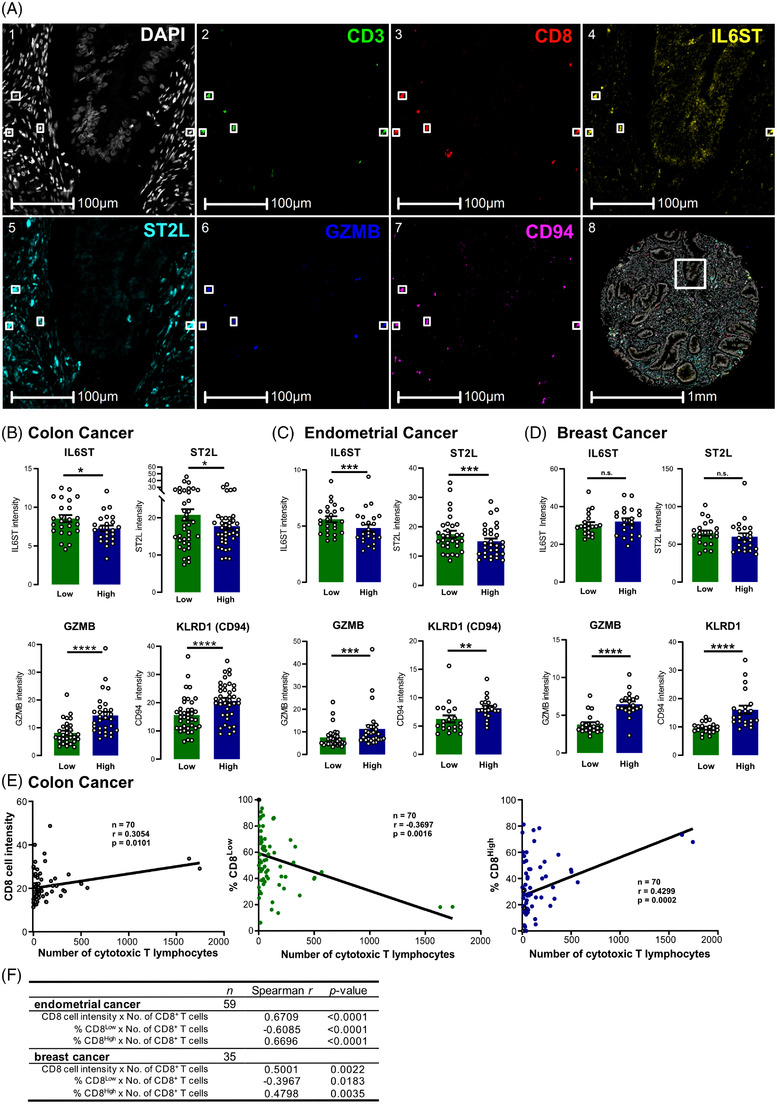
CD8^Low^ and CD8^High^ T lymphocyte subpopulations are present in colon, endometrial and breast cancer patients. (A) Representative immunofluorescence images from a colon cancer TMA with stainings for CD3 (2, green), CD8 (3, red) for cytotoxic T lymphocytes and together with markers of interest IL6ST (CD130) (4, yellow), ST2L (5, cyan), GZMB (6, blue), KLRD1 (CD94) (7, magenta). Nuclei were counterstained with DAPI (1, white). Exemplary cytotoxic T lymphocytes are highlighted in the white boxes. (B–D) Object data of cytotoxic T cells for each patient core was analysed for low (green bars) and high (blue bars) CD8‐expressing T cells and their co‐expression with IL6ST (CD130), ST2L, GZMB and KLRD1 (CD94). Data are shown as mean ± SEM. Quantification in colon cancer patients (*n* = 24 IL6ST, *n* = 38 ST2L, *n* = 30 GZMB, *n* = 39 KLRD1) (B), endometrial cancer patients (*n* = 24 IL6ST, *n* = 30 ST2L, *n* = 28 GZMB, *n* = 20 KLRD1) (C) and breast cancer patients (*n* = 21 IL6ST, *n* = 22 ST2L, *n* = 20 GZMB, *n* = 21 KLRD1) (D), using Wilcoxon matched‐pairs signed rank test. *p ≤ .05, ***p ≤ .001, **** *p* < .0001, n.s., not significant. (E, F) Spearman's correlation of the CD8 cell intensity (black data points), the frequency of CD8^Low^ T cells (green data points) or the frequency of CD8^High^ T cell subset (blue data points) with number of tumour‐infiltrating CD8^+^ T lymphocytes. (E) Representative correlation plots for analysed colon cancer patient TMA cores. (F) Table containing the results from the correlation for endometrial and breast cancer patients

**FIGURE 5:**
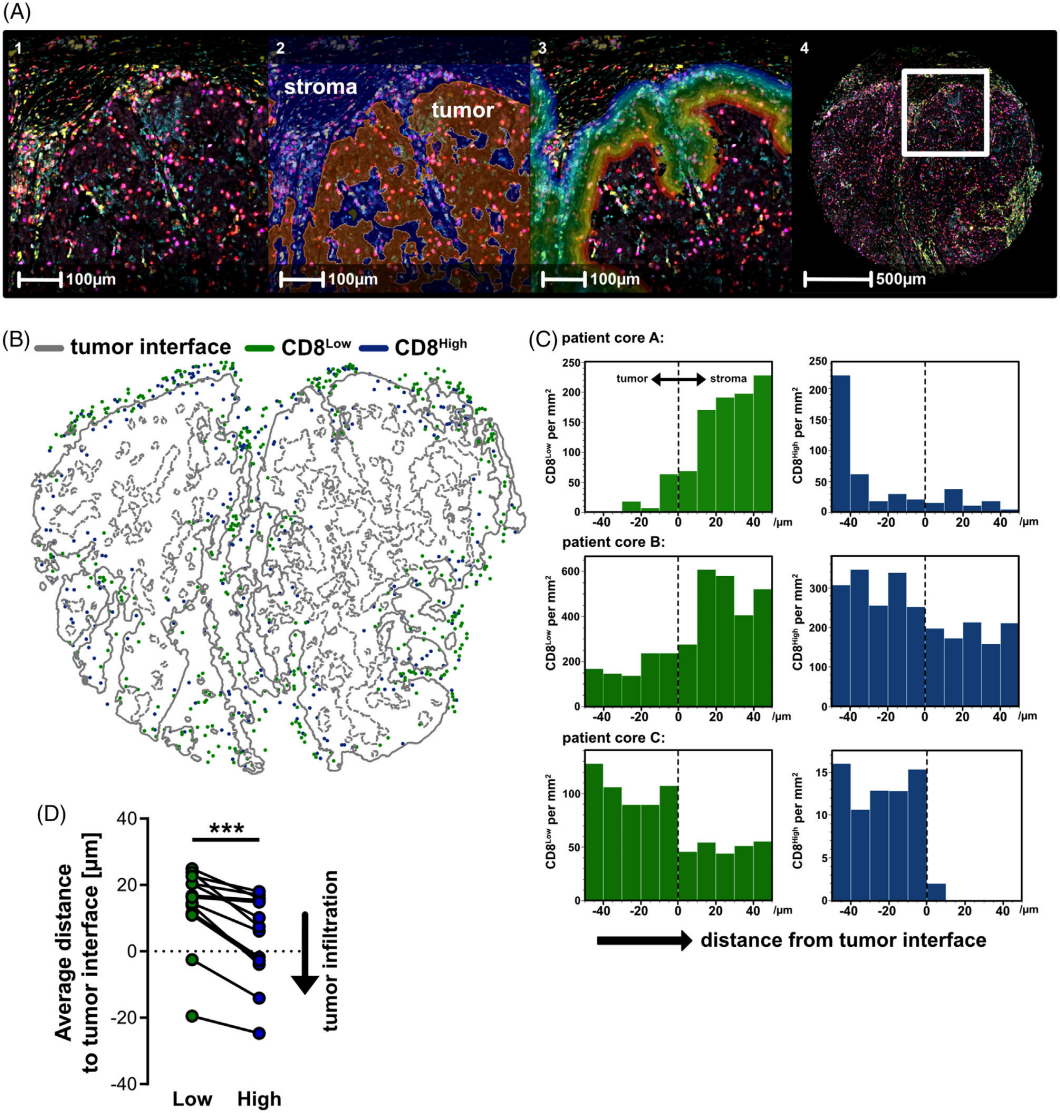
Differential localisation of CD8^Low^ and CD8^High^ T lymphocytes in colon cancer. (A) Fluorescent multiplex images (1) were classified into tumour and stroma compartments (2). The infiltration of CD3^+^ CD8^Low^ and CD8^High^ T cells were then analysed (3) from 50 μm (blue band) to ‐50 μm inside the tumour (red band). (B) Representative spatial analysis plot of tumour‐infiltrating CD8^Low^ (green data points) and CD8^High^ (blue data points). (C) Three representative spatial histograms showing the distribution of CD8^Low^ (green) or CD8^High^ (blue) T cell densities around the tumour interface (0 μm). (D) Quantification of tumour infiltration analysis from *n* = 13 colon cancer patients using Wilcoxon matched‐pairs signed rank test, ***p  <  .001

Next, we investigated whether in vivo correlates of the in vitro generated CD8^Low^ and CD8^High^ T cells existed. Therefore, we analysed tumour microarray (TMA) sections of colon, endometrial and breast cancer patients by PhenOpics multiplex histology (Figure 4A). We had previously connected low CD8 expression by cytotoxic T cells to the induction of the membrane‐bound receptor for IL‐33, suppression of tumourigenicity 2 (ST2)L^14^, ^15^; we included this receptor and IL6ST (CD130) as two promising factors for the validation of CD8^Low^ T cells in tumour sections. Granzyme B as well as KLRD1 (CD94) were used as markers for the CD8^High^ T lymphocytes. CD3 staining was used to preselect for T cells in order to exclude other CD8‐expressing immune cells from the analysis. The number of cytotoxic T cells (CD3^+^ CD8^+^) within the tissue cores was highly heterogeneous as expected. However, we found that low‐CD8‐expressing T cells showed higher IL6ST and ST2L expression in the colon cancer patients (Figure 4B) as well as endometrial cancer patients (Figure 4C), confirming our findings on the CD8^Low^ T lymphocytes upon cell starvation in vitro. Strikingly, CD8^High^ T cells showed a high expression of granzyme B and KLRD1 (Figure 4B–D) in all investigated tumour entities. This underlines the strong link of these effector molecules to CD8^High^ T lymphocytes also in the tumour microenvironment. Next, we analysed whether CD8 expression by cytotoxic T lymphocytes might correlate with differential immune cell infiltration. Notably, we found in all investigated tumour entities that the amount of tumour‐infiltrating cytotoxic T lymphocytes positively correlated with the CD8 intensity (Figure 4E and F, Supplementary Figure S4). Moreover, spatial analysis (Figure 5A) revealed that the CD8^Low^ T lymphocytes accumulated at the tumour margin, whereas the CD8^High^ T cells were localised in close proximity to tumour cells (Figure 5B–D). Taken together, we were able to validate the differential expression of surface markers for both CD8^Low^ and CD8^High^ tumour‐infiltrating T lymphocytes and show a differential localisation of these subsets relative to tumour cells that fits their functional potential.

### 3.5 High frequencies of CD8^Low^ T lymphocytes correlate with cancer cell proliferation and overall tumour progression

**FIGURE 6:**
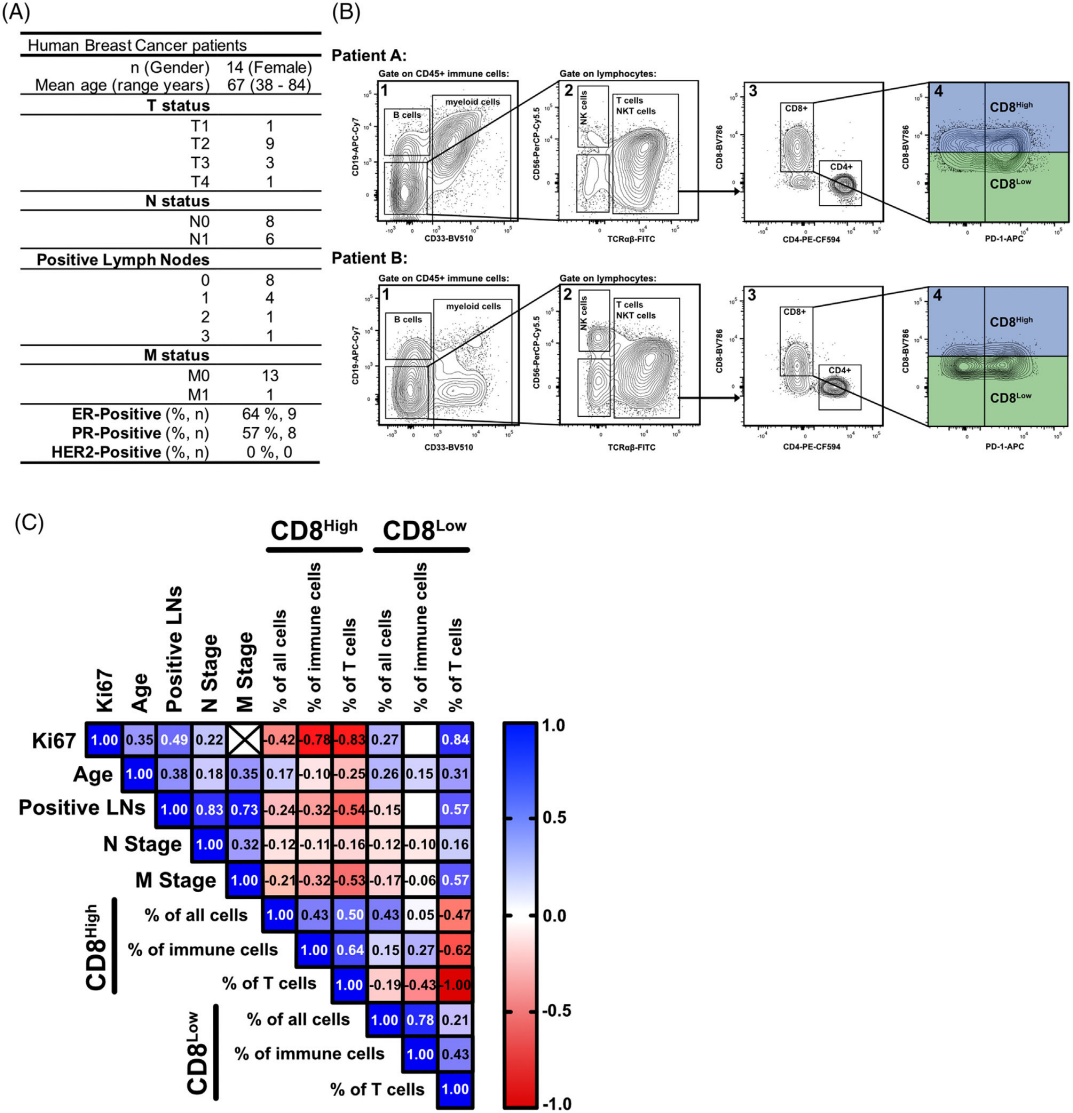
Association of the CD8^Low^ and CD8^High^ T lymphocytes with clinical parameters in mammary carcinoma. (A) Patient characteristics of the breast cancer cohort that was used for the analysis of lymphoid cells. (B) Exemplary FACS plots for the gating of tumour‐infiltrating CD8^Low^ and CD8^High^ T cells derived from two patient samples. (C) Correlation matrix of FACS‐analysed CD8^+^ tumour‐infiltrating T cells and clinical parameters (*n* = 14 patients). A positive correlation is indicated in blue, whereas a negative correlation is shown in red. Numbers indicate the Pearson's correlation coefficient *r*. Blank boxes indicate the absence of correlation (*r* = 0). The cross in the correlation matrix indicates that calculating r between the two variables was not possible

Our analyses so far showed that CD8 expression by itself was a sufficient marker for discriminating phenotypically diverse T cell subsets. We next questioned whether we could find an association between CD8^Low^ or CD8^High^ tumour‐infiltrating T lymphocytes and cancer progression. To this end, we FACS‐analysed lymphocytes from human breast cancer patients (Figure 6A) and assessed the CD8 expression on tumour‐infiltrating cytotoxic T lymphocytes (Figure 6B) in connection to clinical parameters such as Ki67 expression in cancer cells, or the number of affected lymph nodes and distant metastasis as indicators for cancer progression (Figure 6C). Interestingly, high numbers of tumour‐infiltrating CD8^Low^ T lymphocytes were positively correlated with the Ki67 expression of cancer cells, but also with the markers of tumour progression. Thus, CD8 expression by cytotoxic T lymphocytes might be an important indicator of effector function with potential clinical impact by affecting patient prognosis.

### 3.6 Reduction of CD8^Low^ T lymphocytes in the active of relapsing‐remitting multiple sclerosis patients

**FIGURE 7:**
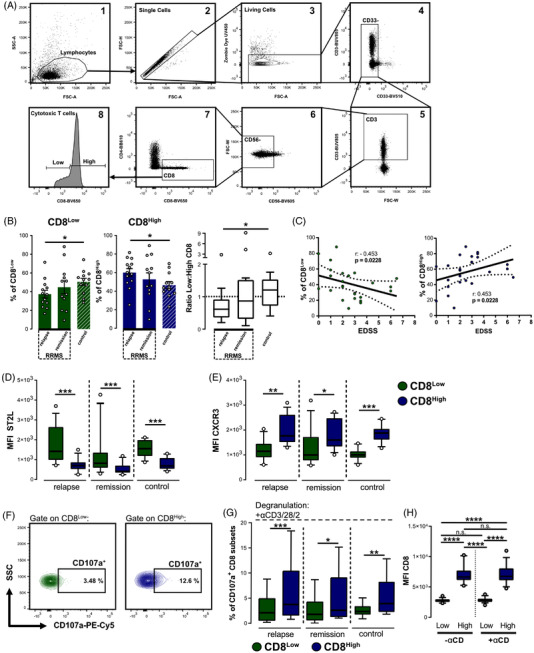
Suppressed CD8^Low^ T cell generation in RRMS patients. CD8^+^ T cells were starved for 40 h as described before and were subsequently analysed by flow cytometry. (A) Gating strategy of CD8^Low^ and CD8^High^ T cell subsets. After identification of the lymphocytes (1), single cells (2) and living cells (3), myeloid cells were excluded (3), as well as CD3^−^ and CD56^+^ immune cells (5, 6). The remaining CD3^+^ CD8^+^ T cells (7) were analysed for their level of CD8 (BV650) expression (8). (B) CD8^Low^ and CD8^High^ T cell frequencies of RRMS patients in relapse (*n* = 13) and in remission (*n* = 12) were compared to healthy controls (*n* = 12). Data are presented as mean ± SEM. Comparisons between the different study groups using the Mann–Whitney *U* test. *p ≤ .05. (C) Spearman's correlation of CD8^Low^ (green data points) or CD8^High^ T cell frequencies with the expanded disability status score (EDSS) for all (*n* = 25) RRMS patients. Dashed lines indicate 95% confidence intervals bands for the regression line. (D) ST2L (FITC) and (E) CXCR3 (APC) mean fluorescence intensities (MFI) of FACS‐analysed CD8^Low^ (green) and CD8^High^ (blue). (F–H) PBMC from the same patient cohort were additionally treated with anti‐CD3/28/2 antibodies and degranulation (CD107a) was assessed by flow cytometry. (F) Representative FACS plots for CD107a^+^ CD8^+^ T cell subsets. (G) Frequencies (%) of CD107a^+^ CD8 T cell subsets. All box plots mark the 5th percentile, the median and 95th percentiles. *p ≤ .05, **p < .01, ***p < .001, using Wilcoxon matched‐pairs signed rank test. (H) Comparisons of CD8 expression (MFI) for the subpopulations with or without TCR stimulation (±αCD), *n* = 37, n.s. not significant, ****p < .0001, using Friedman's test with Dunn´s posttest

Beside their intriguing role within the TME, cytotoxic CD8^+^ T lymphocytes have also been described to play a functionally diverse role in multiple sclerosis.^21^ Therefore, we extended our studies to include RRMS patients. We suspected an imbalance of the regulatory and effector T cell responses which might also become apparent for our different CD8^+^ T cell subpopulations derived from RRMS patients (Figure 7A). Interestingly, in RRMS relapsed patients we saw decreased frequencies of CD8^Low^ T lymphocytes upon starvation indicating a reduced potential to induce this CD8^+^ T cell subset in RRMS. Further, the ratio of CD8^Low^ versus CD8^High^ T lymphocytes analysed by flow cytometry was considerably reduced in the relapsed patients compared to healthy controls (Figure 7B). This reduction in the frequencies of CD8^Low^ T cells upon starvation was found to be negatively correlated with the expanded disability status scale (EDSS) (Figure 7C), a clinical score which reflects disease progression. Again, CD8^Low^ T cells showed enhanced expression of ST2L (Figure 7D), whereas the pro‐inflammatory chemokine receptor CXCR3 was more highly expressed by the CD8^High^ subpopulation (Figure 7E). Accordingly, the measurement of CD8^+^ T cell cytolytic activity in TCR‐stimulated (+αCD3/28/2) samples resulted in higher degranulation of the CD8^High^ T lymphocytes which was observed for all study groups (Figure 7F–H). In summary, upon mTOR‐dependent starvation of cytotoxic T lymphocytes, the population of CD8^Low^ T cells was diminished in the relapsed patients compared to the healthy controls, which might reflect a dominant effector T cell function or the attenuated immunosuppressive regulatory T cell function. Taken together, our data reveal the presence of functionally diverse CD8 low and high‐expressing human cytotoxic T lymphocytes in cancer and RRMS and suggest a differential involvement in disease activity and outcome.

## 4 DISCUSSION

Investigating the immunometabolism of cytotoxic T lymphocytes is an important approach to understand the adaptation of these cells to a dynamic microenvironment and to identify new targets for immune modulation. Despite recent advances in immunotherapy, there is still the challenge to overcome the restriction of potent effector function within the TME.^22^ In addition and as a result of metabolic interference to lymphocytes, current CAR T cell therapy shows only a limited response in solid tumours which emphasises high demand to identify underlying mechanisms to develop novel therapeutic strategies.^23^ Next to an impaired anti‐tumour immunity of cytotoxic T lymphocytes, there is now evidence that also during neuroinflammation the metabolism of immune cells can be strongly affected.^24^ In this current study, we identified two cytotoxic T lymphocyte subpopulations that are marked by their level of CD8 expression which appear to differ in their effector function. Most importantly, these cells are present within the TME but also appear to be dysregulated in RRMS patients.

Although it is known that CD8 expression levels on T cells can change, which eventually modulates the response of the CD8^+^ T lymphocytes.^25^ Other studies on peripheral and circulating CD8^+^ T cells reported decreased CD8 expression being connected to an activated effector phenotype related to viral infections^26^ or to higher expression of suppressive markers in hepatitis B virus infection patients.^27^ In the present study, for the investigated starvation‐induced CD8^Low^ versus CD8^High^ T cell subsets, we observed that the CD8 expression on both sorted subsets was stable after TCR stimulation. Thus, the mTOR inhibition in CD8^+^ T cells upon starvation or rapamycin treatment can induce a lymphocyte subset with decreased CD8 expression and with reduced effector function rather than transiently downregulating CD8. Importantly, the reduction in CD8 expression on T cells is a known mechanism of peripheral tolerance.^13^, ^28^, ^29^

While we found a higher glycolytic demand as well as enhanced expression of T cell effector‐related transcripts (*GZMB*, *KLRD1*, *CXCR3*) in our CD8^High^ T cell subsets, CD8^Low^ appeared to depend less on glycolysis together with a reduced effector molecule expression. We observed CD8^Low^ with enhanced ST2L and IL6ST receptor expression in colon and endometrial cancer patients. Overall, differences in the expression of markers at protein level between the CD8^Low^ and CD8^High^ T cell subsets in these samples were smaller compared to our in vitro data. This might be explained by the complex and heterogeneous situation found in the TME compared to the in vitro system. Moreover, the tyramide‐HRP amplification technology employed in our studies results in labelling epitopes and neighbouring areas with multiple fluorochrome molecules, which likely blunts expression differences. Nevertheless our findings indicate, first of all, the existence of CD8^Low^ T cells within tumour sections that we initially studied under in vitro starvation within tumour sections and opens up perspectives to selectively target these cells. The blockade of IL‐6 signalling is already considered as an additional approach for anti‐cancer therapy,^30^ which may modulate CD8^Low^ T cells as well based on our findings. Moreover, also in line with our findings, the alarmin IL‐33 and ST2 pathway that has been connected to a pro‐tumourigenic role in tumour‐infiltrating immune cells^31^, ^32^ and may be a further strategy to overcome the limited lymphocyte function within the TME. Since a high overall CD8^+^ T cell infiltration accompanied high frequencies of CD8^High^ T cells, which implies a favoured prognosis in the individual patient,^33^ and the number of tumour‐infiltrating CD8^Low^ T cells also correlated with enhanced cancer progression in human breast cancer patients (Figure 5), we suggest that the occurrence of CD8^Low^ and CD8^High^ T cells may be an important predictor or indicator of the patient prognosis. Although this need independent validation, other studies that relate to CD8 expression levels and cancer prognosis in lung adenocarcinoma patients^34^ but also endometrial cancer^35^ support, in principle, our current findings.

The lymphocyte response is an essential component of pathology in autoimmune diseases like multiple sclerosis.^36^ Effector T cell responses that cause the damage in the CNS are known to outweigh the regulatory T cell function that normally protects against the excessive inflammation.^37^, ^38^ In order to reconstitute this disturbed balance in patients, there is substantial need of identifying the underlying pathophysiological mechanisms. CD8^+^ cytotoxic T lymphocytes are found in the CNS of afflicted patients^21^, ^39^ and seem to play an essential role in the pathophysiology of the disease besides other involved immune cells (for review see Ref. ^40^). In the present study, we observed that the differentiation of CD8^+^ T lymphocytes subpopulations under mTOR‐dependent starvation seemed to be dysregulated in RRMS, particularly in patients experiencing relapse. In line with these findings, our previous reports showed the capacity of starved CD8^+^ T lymphocytes to induce the regulatory transcription factor FoxP3^14^ and to suppress the proliferation of human responder T cells.^15^ This emphasises an imbalance between the cytotoxic effector functions and the ability to develop regulatory CD8^+^ T lymphocyte subsets in RRMS patients. Other studies investigated CD8 expression on blood‐derived lymphocytes of MS patients. Interestingly, a NK cell population of CD8^Low^ CD56^+^ CD3^−^ CD4^−^ was described to be diminished in the peripheral blood of RRMS patients in absence of any treatment, reflecting the fact that there exist, in principle, an imbalance also for other immune cells subsets.^41^ Another study reported on the CD8‐dependent T cell subsets in the peripheral blood of MS patients with an unchanged frequency of circulating CD8^Low^ T cell subset in RRMS patients,^42^ which was in contrast to the present study not investigated after starvation. Studying the processes of immunometabolism in the context of neuroinflammation is a current task of the field as the impact of metabolic changes on immune cells found in the CNS and remains largely undescribed.^24^ A number of studies also focus on autophagy as central metabolic pathway in neurodegenerative disorders.^43^ Of note, rapamycin as mTOR inhibitor and potent inducer of autophagy showed favourable effects in the EAE model for MS^44^, ^45^, ^46^, ^47^ as well with RRMS patients.^11^ Thus, rapamycin as well as starvation‐induced mTOR inhibition may limit destructing effector responses, in return promoting CD8^Low^. This further suggests an intriguing role of mTOR modulation for the treatment of autoimmune disease including RRMS. In summary, our data provide the evidence for the involvement of immunological distinct and phenotypically diverse CD8^+^ T lymphocyte subpopulations not only in cancer but also in RRMS patients. However, independent validation using larger patient cohorts will be required. Understanding the molecular mechanisms how the immune cells metabolically adapt to a changing microenvironment may help to improve the development of therapeutic strategies in immune‐related diseases.

## 5 CONCLUSION

In this study, we show that human low‐ and high CD8‐expressing cytotoxic T cells generated upon mTOR inhibition exhibit distinct phenotypes. Cytotoxic T cell populations corresponding to these phenotypes are present in human tumours, where they show divergent localisation relative to tumour cells and particularly the CD8^Low^ T cells are associated with poor prognosis of breast cancer patients. The presence of these subsets extends beyond cancer, since we observed a reduction of CD8^Low^ compared to CD8^High^ T cells particularly in RRMS patients experiencing relapse. Thus, our data indicate an overall immunological relevance of these CD8 subsets, which may open new avenues to specifically target individual CD8 T cell subsets in cancer and autoimmunity.

## CONFLICT OF INTEREST

The authors declare that the research was conducted in absence of any commercial or financial relationship that could be construed as a potential conflict of interest.

## Supporting information

Supporting Information
