# Supplementary material for: Differential expression of CD8 defines phenotypically distinct cytotoxic T cells in cancer and multiple sclerosis
Source: Clin Transl Med. 2022 Dec 11;12(12):e1068. doi: 10.1002/ctm2.1068 (PMC9742381; doi:10.1002/ctm2.1068)
Supplement: Supplementary file 1 — Supporting Information [file CTM2-12-e1068-s001.docx]

**Supplemental Data**

Figure S1

**
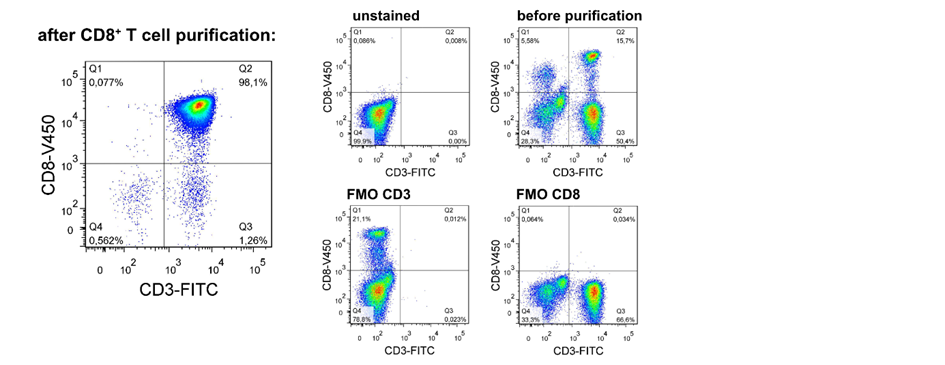
**

**Figure S 1: Representative FACS plots for the purity assessment of human CD3^+^ (FITC) CD8^+^ (V450) T cells.** Human CD3^+^ CD8^+^ T lymphocytes were enriched from PBMC by immunomagnetic negative selection.


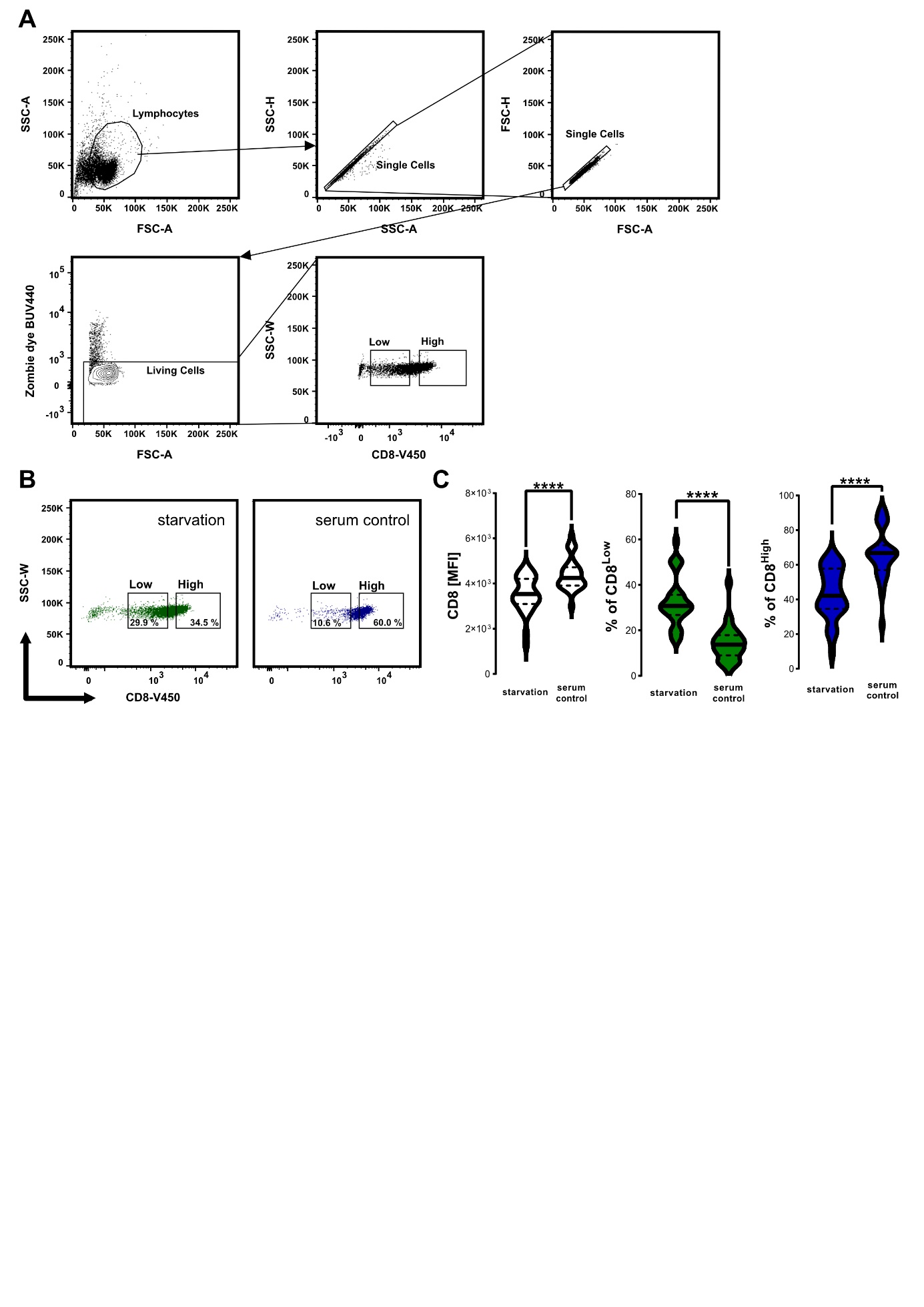
Figure S2

**Figure S 2: Gating strategy of FACS sorted CD8^Low^ and CD8^High^ T lymphocytes. (A)** After identification of single and living cells, low and high CD8-expressing T-cells were separated. **(B)** Gating was controlled by comparing the CD8 expression of starved cytotoxic T lymphocytes to cytotoxic T-cells that were cultured with 10 % autologous donor serum. **(C)** CD8 Mean fluorescence intensity (MFI) (white) as well as frequencies of CD8^Low^ (green) and CD8^High^ T-cells of starved and 10 % serum culture control samples. **** p < 0.0001 using Wilcoxon matched-pairs signed rank test.

Figure S3

**
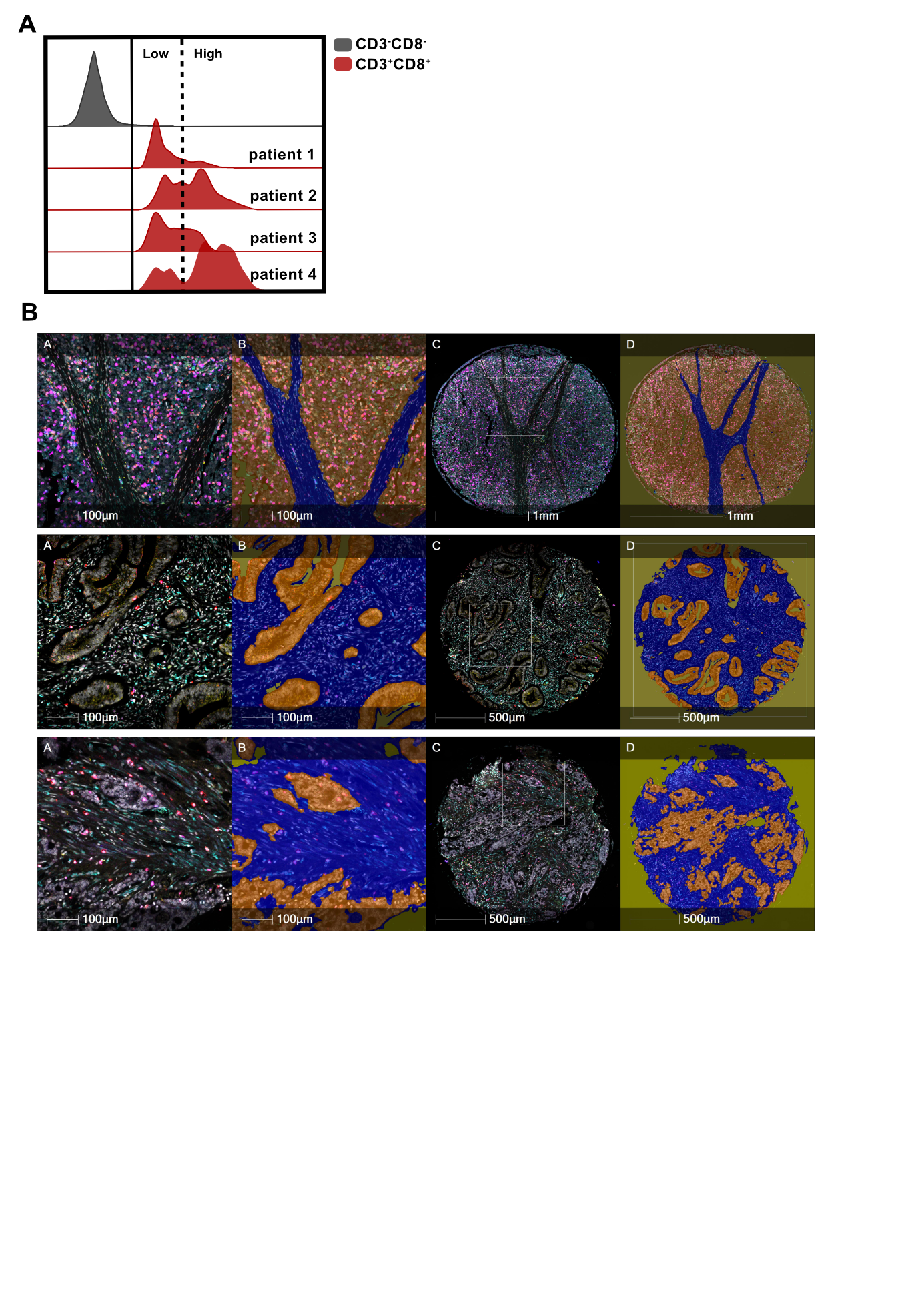
**

**Figure S 3: TMA core analysis. (A)** Exemplary gating of the CD8^Low^ and CD8^High^ subset in FCS.-extracted object data (CD8 intensity) from the HALO image analysis in four different patient cores. **(B)** Random forest algorithm tissue classification (HALO module Classifier) in three exemplary colon cancer cores. Epithelial (tumor) tissue (orange), stromal tissue (blue), empty spots (yellow). The tissue classifier was trained with 3-4 examples given from each class.

Figure S4

**
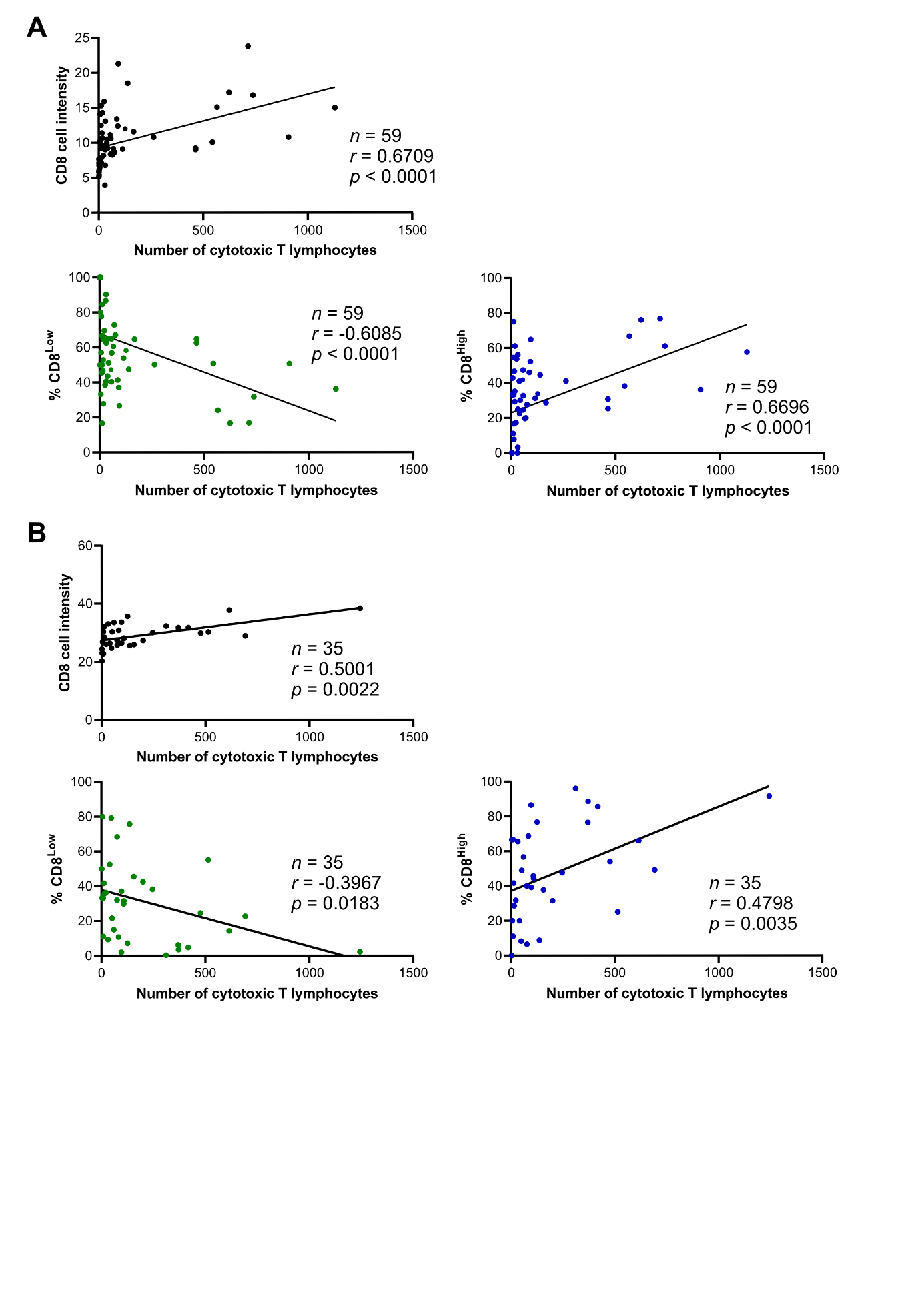
**

**Figure S 4: Spearman correlations in endometrial cancer cores (A) and breast cancer cores (B).** Correlations of CD8 cell intensity of all analyzed cytotoxic T lymphocytes (black data points), the frequency of CD8^Low^ T cells (green data points) or the frequency of CD8^High^ T cell subset (blue data points) with number of tumor-infiltrating CD8^+^ T cells.

Figure S5

**
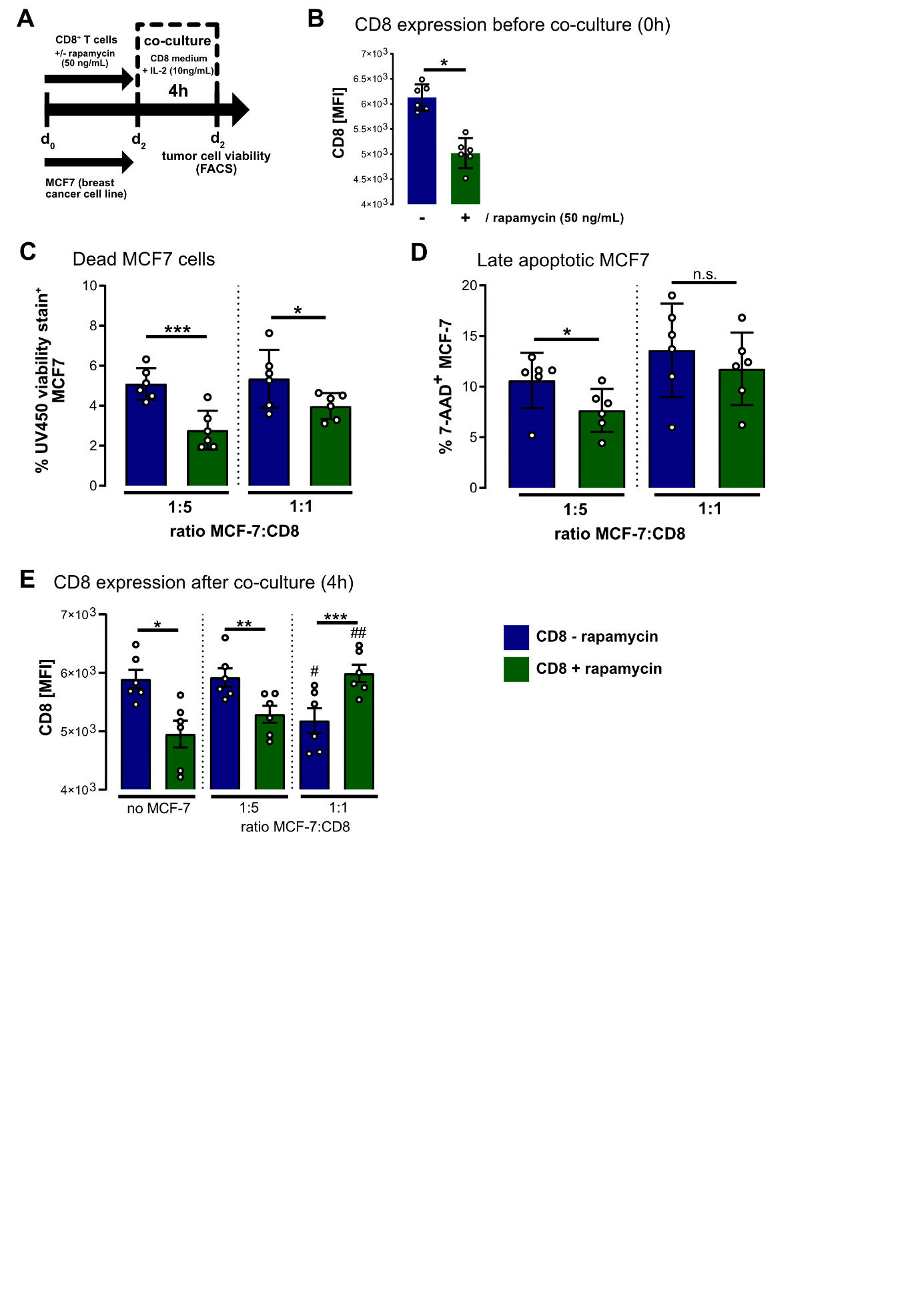
**

**Figure S 5:** Allogenic T cell cytotoxicity assay with human breast cancer MCF7 cells. Purified human CD8^+^ T lymphocytes were treated with 50 ng/mL rapamycin for “pseudo-starvation” and induction of low CD8α expression. 3 x 10^4^ MCF7 cells were plated into 96-well plates 2 h before co-culture. Afterwards, cytotoxic T cells were harvested and added with the ratios (MCF7:CD8) 1:10, 1:5 and 1:1 in order to start the co-culture. CD8 culture medium was supplemented and used for the time of the co-culture. After 4h, the cells were harvested and CD8-targeted tumor cells viability was assessed by flow cytometry measurement. **(A)** Experimental timeline. **(B)** CD8α (V450) mean fluorescence intensity (MFI) expression before co-culture (0h) and **(E)** after 4h. FACS-analyzed of **(C)** the frequency UV450 viability stain+ and **(D)** late apoptosis (7-AAD+) in the MCF7 cell population. The bars show mean ± SEM (n = 6 donors), n.s. not significant, *p ≤ 0.05, **p ≤ 0.01, ***p ≤ 0.001 using the paired t-test. (E) # indicate multiple comparisons to respective control, *p ≤ 0.05, **p ≤ 0.01 using RM one-way ANOVA.

Table S1

**Table S1: Summary of TMA patient cohorts.**

| TMA | Colon Cancer | Endometrial Cancer | Breast Cancer |
| --- | --- | --- | --- |
| Patient cores | *n* = 72 | *n* = 102 | *n* = 107 |
| Intact core/ sufficient core quality | *n* = 70 | *n* = 59 | *n* = 35 |
| CD8^Low/High^ x IL6ST | *n* = 24 | *n* = 24 | *n* = 21 |
| CD8^Low/High^ x ST2L | *n* = 38 | *n* = 30 | *n* = 22 |
| CD8^Low/High^ x GZMB | *n* = 30 | *n* = 28 | *n* = 20 |
| CD8^Low/High^ x KLRD1 | *n* = 39 | *n* = 20 | *n* = 21 |

Table S2

**
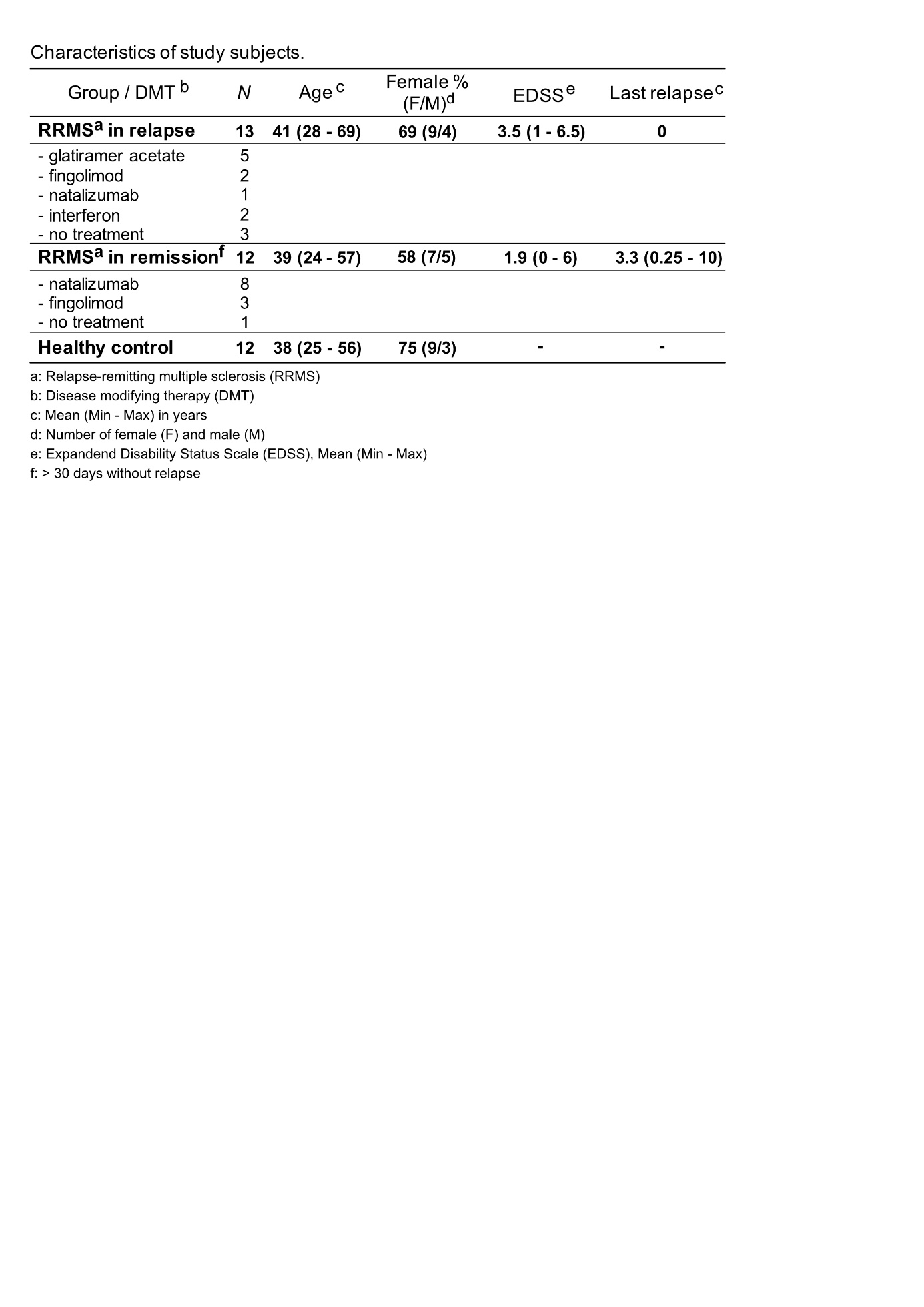
Table S2: Characteristics of RRMS patients.**

**Supplemental Methods**

**Co-Culture of CD8 T cell subsets with human MCF-7 cells**

Human breast cancer cell line MCF7 was cultured in RPMI 1640 + Glutamax medium, which was supplemented with 1 mM sodium pyruvate, 100 μg/mL streptomycin and 100 IU/mL penicillin (all from Thermo Fisher Scientific, Waltham, MA) and 10 % fetal calf serum (FCS) (Merck, Darmstadt, Germany). Human cytotoxic T lymphocytes were purified from human PBMC (for details see section 2.2) and treated with either 50 ng/mL rapamycin (LC Laboratories, Woburn, MA) or a respective DMSO control for 2 days. Before co-culture, 4 x 10^3^ MCF7 cells per well were seeded into 96-well plates. After 2h, CD8^+^ T cells were harvested and added in two MCF7:CD8 ratios (1:5 and 1:1) to the 96-well plate. During co-culture, the cells were cultured in T cell medium (RPMI, section 2.2.) supplemented with 10 ng/mL IL-2 (peprotech, Cranbury, NJ). The allogenic CD8 T cell cytotoxicity was assessed after 4h of direct co-culture by using FACS measurement of the viability of MCF7 cells. To this end, harvested cells were stained with anti-CD8-V450 (clone: RPA-T8, BD Biosciences, Heidelberg, Germany, RRID: AB_1645581), the Zombie UV^TM^ Fixable Viability Kit (BioLegend, San Diego, CA), anti-CD45-AF700 (BD Biosciences, San Diego, CA, RRID: AB_493760) and 7-AAD (BD Biosciences, San Diego, CA, RRID: AB_2869266). A time line of the experimental set-up is also provided in Fig. S 5.
